# Exposure to Antipsychotics in Youths at Clinical High Risk for Psychosis: Low VS High Doses and Their Relevance for Clinical Outcomes

**DOI:** 10.1111/eip.70167

**Published:** 2026-03-22

**Authors:** Lorenzo Pelizza, Emanuela Leuci, Emanuela Quattrone, Alessandro Di Lisi, Derna Palmisano, Simona Pupo, Giuseppina Paulillo, Clara Pellegrini, Pietro Pellegrini, Marco Menchetti

**Affiliations:** ^1^ Department of Biomedical and Neuromotor Sciences Alma Mater Studiorum—Università di Bologna Bologna Italy; ^2^ Department of Mental Health and Pathological Addiction Azienda USL di Parma Parma Italy; ^3^ Pain Therapy Service, Department of Medicine and Surgery Azienda Ospedaliero‐Universitaria di Parma Parma Italy

**Keywords:** antipsychotic, clinical high risk, early intervention in psychosis, early psychosis, follow‐up, outcome

## Abstract

**Introduction:**

Predicting prognosis in subjects at Clinical High Risk for Psychosis (CHR‐P) is still challenging. In particular, there are some disregarded factors such as ongoing Antipsychotic (AP) treatment that potentially induce method errors and research bias. The specific purpose of this examination was to examine whether baseline AP exposure and its dosage identify different CHR‐P groups with diverse prognostic outcomes across 2 years of follow‐up.

**Methods:**

182 CHR‐P participants (93 AP‐naïve, 60 low‐dose AP, 33 high‐dose AP) were assessed for a broad range of clinical outcomes, including psychosis transition, clinical and functional remission (measured with the Positive and Negative Syndrome Scale and the Social and Occupational Functioning Assessment Scale). Inter‐group comparisons were explored using Kaplan–Meier survival analyses and binary logistic regression analyses.

**Results:**

Across the follow‐up, the high‐dose AP subgroup showed an increased risk of new hospitalisation and poorer functional remission compared to AP‐naïve participants. Conversely, the low‐dose AP subsample had a higher 2‐year rate of symptomatic remission and a lower 2‐year rate of self‐harm behaviour compared to AP‐naïve one.

**Conclusion:**

APs at low doses are associated with better symptomatic outcomes, whilst APs at high doses correlate to poorer socio‐occupational functioning. Promoting personalised treatment strategies and facilitating the identification of specific CHR‐P subgroups with divergent prognoses are recommended in clinical practise.

## 1 Introduction

Predicting prognosis in individuals at ‘Clinical High Risk for Psychosis’ (CHR‐P) is still a research challenge despite different models focused on several baseline parameters (Zhu et al. 2024; Haas et al. 2024). In fact, selective bias resulting from poor consideration of some poorly examined confounders, such as ongoing *Antipsychotic* (AP) therapy, can potentially influence outcome results and compromise the precision of the method (Raballo et al. 2025), albeit their effects are difficult to quantify precisely (Raballo et al. 2024a).

In this regard, relatively recent meta‐analysis results suggested that CHR‐P subjects with AP prescription at presentation in ‘Early Intervention in Psychosis’ (EIP) services had higher incidence rates of psychosis conversion over time compared to peers without AP exposure, independently from overall population size, age, sample enrichment, gender ratio, and quality of the investigations (Raballo et al. 2020, 2024b). Moreover, they showed other remarkable clinical differences in terms of both baseline features (e.g., older age, more severe psychopathology, and lower daily functioning levels) (Zeng et al. 2022; Zhang, Raballo, et al. 2022; Pelizza et al. 2024) and poorer prognostic outcomes (e.g., higher incidence rates of new hospitalisation, worst cognitive recovery) (Zhang, Wang, et al. 2022; Pelizza, Di Lisi, Leuci, et al. 2025).

Although evidence on actual beneficial effects of AP treatment in preventing psychosis and ameliorating longitudinal outcomes in CHR‐P subjects is limited (Catalan et al. 2020; Poletti et al. 2024), clinicians' decision‐making on AP exposure in youths at CHR‐P is more commonly related to higher perceived clinical severity and functioning disability at presentation (Di Lisi, Leuci, et al. 2024). In this respect, AP prescription may be considered a ‘need‐based’ pharmacological indication motivated by a global apprehension for the ongoing, highly severe psychopathological process requiring non‐deferrable AP treatment (Preti et al. 2022). This inevitably impacts on clinical outcomes and psychosis conversion risk assessment in CHR‐P populations, and as suggested by Raballo et al. (2021), we could refine the prognostic staging of CHR‐P mental states with clinically pragmatic anchor points along a gradient of progressive care needs, which are parallel to the risk of incurring in severe outcomes: i.e., AP‐naive CHR‐P, CHR‐P in low‐dose AP monotherapy, and CHR‐P in high‐dose AP mono‐therapy or AP poly‐therapy. In this regard, ongoing AP treatment at baseline could clinically mask an already‐developed psychosis, making these cases incorrectly identified as CHR‐P subjects, rather than CHR‐P with a higher risk of transition (Raballo et al. 2025).

Starting from this background, the primary *goal* of this investigation was to discuss whether AP exposure really detects a CHR‐P subgroup with poorer outcomes, especially high‐dose AP individuals. Specifically, we analysed a broad range of outcome parameters together with psychosis conversion comparing them amongst three different CHR‐P subsamples (i.e., AP‐naïve, low‐dose AP‐exposed, and high‐dose AP‐exposed) enrolled within an Italian EIP service across 2 years of follow‐up.

## 2 Materials and Methods

### 2.1 Subjects and Setting

All our CHR‐P youths were recruited within the ‘Parma At‐Risk Mental States’ (*PARMS*) programme between 1st January 2016 and 31st December 2021. This EIP protocol was widely implemented in all adult and child/adolescent adult mental healthcare services of the Parma Department of Mental Health (Northern Italy) (Pelizza et al. 2023).

*Inclusion criteria* were: (a) specialist (mental health assistance) request, (b) age 12–25 years, and (c) to meet CHR‐P criteria as described by the ‘Comprehensive Assessment of At‐Risk Mental States’ (CAARMS) criteria at entry: i.e., ‘Vulnerability Group’ (VG), ‘Brief Limited Intermittent Psychotic Symptoms’ (BLIPS), and ‘Attenuated Psychotic Symptoms’ (APS) (Yung et al. 2005).

*Exclusion criteria* were: (a) past clinically evident psychotic episode; (b) past AP exposure (i.e., in previous illness episode) or current AP exposure exceeding 4 weeks in the current episode; (c) medical disease with psychopathological manifestations; and (d) known intellectual disability (i.e., IQ < 70). Past AP exposure was intended as a proxy for a past psychotic phase, consistently with the original CAARMS definition of psychosis threshold (Raballo et al. 2019). A current AP exposure of < 4 weeks was mandatory to minimise psychopharmacological interference with baseline clinical evaluation, as defined in the PARMS procedures (Biancalani et al. 2025).

Following the present official EIP guidelines (Barnes et al. 2020; GLR 2024), AP prescription at presentation was reserved when CHR‐P youths had sudden functioning decline, rapid escalation of psychotic features, high risk of suicide or violence, or failed to respond to psychosocial treatments.

This investigation obtained local ethical approvals (‘Area Vasta Emilia Nord’ ethics committee protocol no. 559/2020/OSS*/AUSLPR) and was conducted in accordance with the 1964 Helsinki declaration and its later amendments. All subjects (including minors and their guardians) provided their written informed consent for their participation in the research.

### 2.2 Assessment

For the specific purposes of this examination, the psychopathological evaluation included the CAARMS and the Positive and Negative Syndrome Scale (PANSS) (Kay et al. 1987).

The *CAARMS* is a clinical interview to examine the multifaceted features of attenuated psychopathology. It specifically defines both CHR‐P and psychosis threshold criteria using its ‘Positive Symptoms’ subscale scores. The CAARMS was administered by trained PARMS staff members using the authorised Italian version (CAARMS‐ITA) (Pelizza et al. 2019). CAARMS supervisions and scoring workshops were regularly conducted over time to ensure good interrater reliability values (Paterlini et al. 2019).

The CAARMS also measured socio‐occupational functioning through the integrated Social and Occupational Functioning Assessment Scale (*SOFAS*) module. It showed good psychometric properties in Italian CHR‐P samples (Di Lisi, Leuci, et al. 2024). In this examination, a current SOFAS score of > 60 at follow‐ups was considered as an index of functional remission (Schennach‐Wolff et al. 2009).

The *PANSS* is a clinical interview to assess psychopathology in patients with psychosis, including its early stage (Ricci et al. 2024). In this investigation, trained PARMS staff members administered the approved Italian version of the PANSS (Pancheri et al. 1995) and used a score of ≤ 3 on the 8 PANSS items designated in the Remission in Schizophrenia Working Group's criteria as symptomatic remission indicator (Andreasen et al. 2005). As additional relevant outcome, we also evaluated the longitudinal course of persistent negative symptoms, defined in accordance with the more conservative criteria proposed by Buchanan (2007): i.e., (1) at least ‘moderate’ (score of ≥ 4 on the PANSS) for at least 3 negative symptoms or at least ‘moderately severe’ (score of ≥ 5 on the PANSS) for at least 2 negative symptoms; (2) persistence of symptoms for at least 6 months and for an extended period of time (at least 4 weeks) prior to the enrollment; and (3) absence of clinically relevant levels of positive symptoms, depression and extrapyramidal symptoms, as defined thresholds on validated rating instruments. The latter criterion was intended as persistent score of ≤ 3 in all the PANSS items included in the ‘Positive Symptoms’ dimension and PANSS ‘Depression’ and ‘Guilt Feelings’ items, together with the absence of extrapyramidal features requiring anticholinergic medication (Pelizza et al. 2022).

The clinical assessment was repeated every 12 months along the follow‐up period, and CAARMS criteria were checked to detect a possible psychosis conversion and the persistence of a CHR‐P status. Additionally, a *clinical chart* including information on service disengagement, new attempted suicide, new self‐harm behaviour, new hospital admission, and functional recovery was also completed every year during the follow‐up (see Table S1 for details on the definitions of these outcome parameters).

### 2.3 Procedures

After administering the CAARMS, CHR‐P individuals without AP prescription at entry were classified as ‘AP‐naïve’, whilst those with AP exposure were grouped in accordance with their AP dose (i.e., ‘high‐dose’ or ‘low‐dose’ CHR‐P/AP+ subgroups). Specifically, based on the method proposed by Roh et al. (2014), we used the ‘Prescribed Daily Dose’ (PDD)/‘Defined Daily Dose’ (DDD) ratio to define AP dosage and categorisation. The PPD was calculated as risperidone equivalent dose in milligrammes (Catalano et al. 2025). The DDD was defined as the assumed average maintenance dose per day for a drug used for its main indication in adults (Leucht et al. 2016). It is the international unit approved by the World Health Organization (WHO) for drug use studies (WHO 2002). According to Taipale et al. (2022), a PDD/DDD of ≤ 0.6 (i.e., less than 0.6 defined daily doses per day, corresponding to 3 mg equivalent risperidone per day) was considered as the cut‐off value for low dose.

After CAARMS interviews, all participants were then assigned to PARMS multi‐professional teams including clinical psychologists, case managers for early rehabilitation, and psychiatrists, generally within 4 weeks. According to current EIP guideline recommendations (Di Lisi, Pupo, et al. 2024), AP exposure at entry had to be reserved for CHR‐P subjects who (1) experienced abruptly worsening psychotic features, (2) showed a sudden functioning decline, (3) had an increased risk of suicide or violence, or (4) did not respond to first‐line psychosocial interventions. Outcome information was collected every 12 months across the follow‐up.

### 2.4 Statistical Analysis

Collected data were examined using the Statistical Package for Social Science (SPSS) version 28.0 for Windows (IBM Corp. 2021). All tests were two‐tailed with a significance *p* level set at 0.05. Bonferroni correction was calculated to deal with multiple comparisons and family‐wise error rates (Armstrong 2014).

As for inter‐group comparisons on time‐to‐event outcome parameters (e.g., service disengagement, new suicide attempt), Kaplan–Meier survival analysis was used (Goel et al. 2010), whilst for not time‐to‐event dependent variables (e.g., suicidal ideation, functional recovery) binary logistic regression analysis with CHR‐P subgroup as independent measure was carried out (Harris 2021).

## 3 Results

A total of 182 CHR‐P individuals were enrolled in this investigation (91 [50%] males, 112 [89.6%] white Caucasian, mean age = 19.55 ± 3.79 years old). Ninety‐three (51.1%) of them showed an AP prescription at entry (60 [33.0%] at ‘low dose’ and 33 [18.1%] at ‘high dose’), whilst the remaining 89 (48.9%) were included in the ‘AP‐naïve’ subgroup. At presentation, AP‐naïve participants were younger compared to the other two CHR‐P subsamples (Kruskal‐Wallis test *H* value = 21.484; *p* = 0.001) and had a lower prevalence rate of BLIPS (*X* ^2^ test value = 9.406; *p* = 0.009) and a higher SOFAS score in comparison with high‐dose subjects (Kruskal‐Wallis test *H* value = 6.985; *p* = 0.030) (see also Table S2 for details on baseline sociodemographic and clinical characteristics amongst the three subgroups).

One hundred and fifty‐three (84.1%) CHR‐P participants concluded the 2‐year follow‐up period (70 [78.6%] AP‐naïve, 54 [90.0%] low‐dose, and 29 [87.9%] high dose) (see also Figure S1 for details). As for outcome parameters, AP‐naïve individuals showed a lower 2‐year incidence rate of new hospitalisation compared to the other two subgroups and a higher incidence rate of new self‐harm behaviour in comparison with low‐dose subjects (Table 1) (see also Figure S2 for details on survival functions in the three subsamples). No inter‐group differences in terms of service disengagement, new attempted suicide, and psychosis transition were found.

**TABLE 1:** Kaplan–Meier survival analysis results: Comparisons on 2‐year time‐to‐event outcome incidence rates amongst the three CHR‐P subgroups.

CHR‐P subgroup	Number of events	1‐cumulative proportion surviving at the time		Mean (in months) for 2‐year *service disengagement* incidence rate			
		Estimate	SE	Estimate	SE	95% CI	
						Lower bound	Upper bound
AP‐naïve	19	0.213	0.043	21.146	0.595	19.980	22.312
Low‐dose	6	0.100	0.039	22.800	0.465	21.889	23.711
High dose	4	0.121	0.057	22.333	0.803	20.760	23.906
Overall	29	—	—	21.907	0.364	21.193	22.620

Log rank (Mantel‐Cox)	*Χ* ^2^	df	*p*
Low‐dose vs. AP‐naïve	3.478	1	0.062
High‐dose vs. AP‐naïve	1.283	1	0.257
High dose vs. low dose	0.127	1	0.722

CHR‐P subgroup	Number of events	1‐cumulative proportion surviving at the time		Mean (in months) for 2‐year *new hospitalisation* rate			
		Estimate	SE	Estimate	SE	95% CI	
						Lower bound	Upper bound
AP‐naïve	6	0.071	0.028	23.143	0.337	22.482	23.804
Low‐dose	13	0.219	0.054	21.600	0.645	20.336	22.864
High dose	7	0.223	0.074	21.750	0.894	19.997	23.503
Overall	26	—	—	22.364	0.317	21.743	22.984

Log Rank (Mantel‐Cox)	*Χ* ^2^	df	*p*
Low‐dose vs. AP‐naïve	6.362	1	**0.012**
High‐dose vs. AP‐naïve	4.967	1	**0.026**
High dose vs. low dose	0.999	1	0.987

CHR‐P subgroup	Number of events	1‐cumulative proportion surviving at the time		Mean (in months) for 2‐year *new suicide attempt* incidence rate			
		Estimate	SE	Estimate	SE	95% CI	
						Lower bound	Upper bound
AP‐naïve	7	0.089	0.032	23.286	0.335	22.630	23.942
Low‐dose	3	0.054	0.031	23.800	0.243	23.324	24.276
High dose	3	0.094	0.052	22.875	0.618	21.663	24.087
Overall	13	—	—	23.386	0.207	22.980	23.793

Log rank (Mantel‐Cox)	*Χ* ^2^	df	*p*
Low‐dose vs. AP‐naïve	0.705	1	0.401
High‐dose vs. AP‐naïve	0.024	1	0.876
High dose vs. low dose	0.674	1	0.412

CHR‐P subgroup	Number of events	1‐cumulative proportion surviving at the time		Mean (in months) for 2‐year *psychosis transition* incidence rate			
		Estimate	SE	Estimate	SE	95% CI	
						Lower bound	Upper bound
AP‐naïve	11	0.142	0.040	23.000	0.380	22.256	23.744
Low‐dose	11	0.186	0.051	22.200	0.580	21.063	23.337
High dose	7	0.224	0.075	22.125	0.832	20.494	23.756
Overall	29	—	—	22.568	0.298	21.983	23.153

Log rank (Mantel‐Cox)	*Χ* ^2^	df	*p*
Low‐dose vs. AP‐naïve	6.420	1	0.423
High‐dose vs. AP‐naïve	1.188	1	0.276
High dose vs. low dose	0.153	1	0.696

CHR‐P subgroup	Number of events	1‐cumulative proportion surviving at the time		Mean (in months) for 2‐year *new self‐harm behaviour* incidence rate			
		Estimate	SE	Estimate	SE	95% CI	
						Lower bound	Upper bound
AP‐naïve	13	0.164	0.042	22.714	0.421	21.888	23.540
Low‐dose	3	0.050	0.028	23.400	0.338	22.738	24.062
High dose	4	0.125	0.058	22.500	0.702	21.125	23.875
Overall	20	—	—	22.909	0.267	22.386	23.432

Log rank (Mantel‐Cox)	*Χ* ^2^	df	*p*
Low‐dose vs. AP‐naïve	3.929	1	**0.047**
High‐dose vs. AP‐naïve	0.168	1	0.682
High dose vs. low dose	1.652	1	0.199

*Note:* Statistically significant *p* values are in bold. Bonferroni's corrected *p* values are reported.

Abbreviations: AP = Antipsychotic; CHR‐*P* = Clinical High Risk for Psychosis; AP naive = CHR‐P participants without baseline AP prescription; PDD = Prescribed daily dose of AP; DDD = Defined daily dose of AP; Low‐dose = CHR‐P individuals with PDD/DDD ratio < 0.6 (i.e., less than 0.6 defined daily doses per day, corresponding to 3.0 mg per day equivalent risperidone); High‐dose = CHR‐P individuals with a PDD/DDD ratio ≥ 0.6. SE = Standard Error; 95% CI = 95% Confidence Intervals; Log Rank = Logarithm Rank Test; X2 = Chi‐Square test; df = degrees of freedom; *p* = statistical value.

Furthermore, our binary logistic regression analysis results for not time‐to‐event outcome parameters showed that high dose condition at entry was associated to a lower 2‐year incidence rate of functional remission, as well as low dose condition at baseline was related to a higher 2‐year incidence rate of PANSS symptomatic remission, especially when compared to AP‐naïve individuals (Table 2) (see also Figure S3 for details on estimated plots). No between‐group differences in terms of suicidal ideation and CHR‐P criteria persistence were observed.

**TABLE 2:** Binary logistic regression analysis results for not time‐to‐event outcome variables in the three CHR‐P subgroups across the 2‐year follow‐up period.

Dependent variable	AP‐naïve T1 (*n* = 85) T2 (*n* = 70)	Low‐dose CHR‐P/AP+ T1 (*n* = 60) T2 (*n* = 54)	High‐dose CHR‐P/AP+ T1 (*n* = 32) T2 (*n* = 29)	Statistic test			
				*X* ^2^	df	*R* ^2^	*p*
1‐year current suicidal ideation	30 (35.3%)	20 (33.3%)	13 (40.6%)	0.485	2	0.003	0.785
2‐year current suicidal ideation	33 (47.1%)	17 (31.2%)	10 (34.5%)	3.481	2	0.022	0.175

1‐year						2‐year				
Current suicidal ideation (yes)	*B* (SE)	OR	95% CI		*p*	*B* (SE)	OR	95% CI		*p*
			Lower	Higher				Lower	Higher	
Low‐dose CHR‐P	0.087 (0.356)	1.091	0.543	2.191	0.807	0.663 (0.378)	1.941	0.925	4.075	0.080
High‐dose CHR‐P	−0.227 (0.426)	0.797	0.346	1.836	0.594	0.527 (0.458)	1.695	0.690	4.160	0.250

Dependent variable	AP‐naïve	Low‐dose	High‐dose	Statistic test			
	T1 (*n* = 85)	CHR‐P/AP+	CHR‐P/AP+	*X* ^2^	df	*R* ^2^	*p*
	T2 (*n* = 70)	T1 (*n* = 60)	T1 (*n* = 32)				
		T2 (*n* = 54)	T2 (*n* = 29)				
1‐year functional recovery	56 (65.8%)	33 (55.0%)	15 (46.9%)	3.995	2	0.022	0.136
2‐year functional recovery	49 (70.0%)	39 (72.2%)	14 (48.3%)	5.273	2	0.034	0.**049**

1‐year						2‐year				
Functional recovery (no)	*B* (SE)	OR	95% CI		*p*	B (SE)	OR	95% CI		*p*
			Lower	Higher				Lower	Higher	
Low‐dose CHR‐P	0.457 (0.346)	1.580	0.802	3.113	0.186	−0.108 (0.400)	0.897	0.409	1.967	0.787
High‐dose CHR‐P	0.783 (0.422)	2.189	0.958	5.001	0.063	0.916 (0.454)	2.500	1.027	6.087	**0.044**

Dependent variable	AP‐naïve	Low‐dose	High‐dose	Statistic test			
	T1 (*n* = 85)	CHR‐P/AP+	CHR‐P/AP+	*X* ^2^	df	*R* ^2^	*p*
	T2 (*n* = 70)	T1 (*n* = 60)	T1 (*n* = 32)				
		T2 (*n* = 54)	T2 (*n* = 29)				
1‐year SOFAS functional remission	55 (64.7%)	34 (56.7%)	17 (53.1%)	1.691	2	0.010	0.429
2‐year SOFAS functional remission	48 (68.6%)	37 (68.5%)	14 (48.3%)	4.081	2	0.026	0.130

1‐year						2‐year				
SOFAS functional remission (no)	*B* (SE)	OR	95% CI		*p*	*B* (SE)	OR	95% CI		*p*
			Lower	Higher				Lower	Higher	
Low‐dose CHR‐P	0.338 (0.346)	1.402	0.712	2.760	0.328	0.002 (0.390)	1.002	0.476	2.153	0.995
High‐dose CHR‐P	0.481 (421)	1.618	0.709	3.690	0.253	0.849 (0.452)	2.338	0.964	5.670	0.060

Dependent variable	AP‐naïve	Low‐dose	High‐dose	Statistic test			
	T1 (*n* = 85)	CHR‐P/AP+	CHR‐P/AP+	*X* ^2^	df	*R* ^2^	*p*
	T2 (*n* = 70)	T1 (*n* = 60)	T1 (*n* = 32)				
		T2 (*n* = 54)	T2 (*n* = 29)				
1‐year PANSS symptomatic remission	45 (52.9%)	34 (56.7%)	19 (59.4%)	0.452	2	0.003	0.798
2‐year PANSS symptomatic remission	46 (65.7%)	46 (85.2%)	23 (79.3%)	6.642	2	0.042	**0.036**

1‐year						2‐year				
PANSS symptomatic remission (no)	*B* (SE)	OR	95% CI		*p*	*B* (SE)	OR	95% CI		*p*
			Lower	Higher				Lower	Higher	
Low‐dose CHR‐P	−0.150 (0.339)	0.860	0.442	1.673	0.657	−1.099 (0.458)	0.333	0.136	0.819	0.**017**
High‐dose CHR‐P	−0.262 (0.420)	0.770	0.338	1.755	0.534	−0.693 (0.523)	0.500	0.179	1.394	0.185

Dependent variable	AP‐naïve	Low‐dose	High‐dose	Statistic test			
	T1 (*n* = 85)	CHR‐P/AP+	CHR‐P/AP+	*X* ^2^	df	*R* ^2^	*p*
	T2 (*n* = 70)	T1 (*n* = 60)	T1 (*n* = 32)				
		T2 (*n* = 54)	T2 (*n* = 29)				
1‐year persistent negative symptoms	7 (8.2%)	9 (15.0%)	5 (15.6%)	2.108	2	0.012	0.349
2‐year persistent negative symptoms	7 (10.0%)	7 (13.0%)	4 (14.0%)	0.402	2	0.003	0.818

1‐year						2‐year				
Persistent negative symptoms (no)	*B* (SE)	OR	95% CI		*p*	*B* (SE)	OR	95% CI		*p*
			Lower	Higher				Lower	Higher	
Low‐dose CHR‐P	−0.676 (0.535)	0.509	0.178	1.452	0.206	−0.293 (0.568)	0.746	0.245	2.272	0.606
High‐dose CHR‐P	−0.724 (0.627)	0.485	0.142	1.655	0.248	−0.365 (0.670)	0.649	0.187	2.581	0.586

Dependent variable	AP‐naïve	Low‐dose	High‐dose	Statistic test			
	T1 (*n* = 85)	CHR‐P/AP+	CHR‐P/AP+	*X* ^2^	df	*R* ^2^	*p*
	T2 (*n* = 70)	T1 (*n* = 60)	T1 (*n* = 32)				
		T2 (*n* = 54)	T2 (*n* = 29)				
1‐year CHR‐P criteria persistence	32 (37.6%)	29 (48.3%)	18 (58.1%)	4.275	2	0.024	0.118
2‐year CHR‐P criteria persistence	22 (31.5%)	17 (31.2%)	12 (40.0%)		2		

1‐year						2‐year				
CHR‐P criteria persistence (no)	*B* (SE)	OR	95% CI		*p*	*B* (SE)	OR	95% CI		*p*
			Lower	Higher				Lower	Higher	
Low‐dose CHR‐P	−0.438 (0.342)	0.645	0.330	1.261	0.200	−0.002 (0.390)	0.998	0.464	2.143	0.995
High‐dose CHR‐P	−0.830 (0.427)	0.436	0.189	1.008	0.052	−0.375 (0.453)	0.688	0.283	1.670	0.408

*Note:* Statistically significant *p* values are in bold. Bonferroni corrected *p* values are reported. Cumulative incidence rates are also reported.

Abbreviations: 95% CI = 95% confidence intervals for OR; AP = antipsychotic; AP naive = CHR‐P participants without baseline AP prescription; B = regression coefficient; CHR‐*P* = Clinical High Risk for Psychosis; DDD = Defined daily dose of AP; df = degree of freedom; High‐dose = CHR‐P individuals with a PDD/DDD ratio ≥ 0.6; Low‐dose = CHR‐P individuals with PDD/DDD ratio < 0.6 (i.e., less than 0.6 defined daily doses per day, corresponding to 3.0 mg per day equivalent risperidone); OR = odds ratio; PANSS = positive and negative syndrome scale; PDD = prescribed daily dose of AP; *R* ^2^ = Cox & Snell's *R* ^2^; SE = standard error; SOFAS = Social and Occupational functioning assessment scale; *X* ^2^ = Chi‐square (*X* ^2^) test value.

## 4 Discussion

The main aim of this examination was to investigate the potential utility of enriching the prognosis prediction in CHR‐P youths by classifying them based on their increasing need for AP treatment: i.e., AP‐naïve, low‐dose AP, and high‐dose AP. Specifically, we analysed a broad range of clinical *outcomes* beyond psychosis conversion across 2 years of follow‐up.

The results of this investigation confirm that a *large portion* (51.1%) of CHR‐P individuals had an AP exposure at entry, with about one in five cases (18.1%) at high doses. In fact, baseline AP prevalence rate varies widely across countries, ranging from 6% reported in the NAPLS‐2 (‘North American Prodrome Longitudinal Study‐2’) (Carrión et al. 2016) to 79% found in the SOPRES (‘Taiwan's Study On the psychopathologic PRogress of Early Schizophrenia‐like disorder’) study (Liu et al. 2011). This could imply different clinical practises across investigation and different cultural attitudes towards medications (Pelizza, Di Lisi, Leuci, et al. 2025), despite current recommendations of official EIP guidelines that advocated not to offer AP as the primary approach to prevent conversion to overt psychotic episode (GAPPP 2019). Additionally, the British Association for Psychopharmacology further specified that if APs are considered for clinical relief, this should be thought of as off‐label prescribing and treated as a short‐term, individual trial (Barnes et al. 2020). However, low AP doses should always be used (Addington et al. 2017). In this regard, it is important to recognise that international guidelines regarding the use of APs in CHR‐P samples may vary depending on the recruitment context and research setting. Indeed, regions that recruit CHR‐P individuals through population‐level screening may show much lower AP prescription rates than tertiary hospital settings, where participants often present with more severe symptoms and actively seek help (Tognin et al. 2025). In such clinical settings, the use of APs tends to be more common and may reflect standard local practises.

In our population at entry, about a fifth of the participants received APs at *high doses* and this drug needs appeared to be in relation to a greater clinical and functioning severity (i.e., higher baseline prevalence rate of BLIPS and higher SOFAS scores) perceived by the treating staff in comparison with AP‐naïve subjects, especially in older ones. In this regard, as the PARMS programme is a specialised EIP protocol, this finding may reflect local practises comparable to those of tertiary specialist settings, in which individuals can often present with more severe CHR‐P symptoms and actively seek help. Indeed, as for AP treatment, our current regional guidelines on interventions in CHR‐P individuals (GLR 2024) state that the clinical decision to prescribe APs should consider the possible presence of rapid functional deterioration, a high risk of self‐harm or other harmful behaviours, and the ineffectiveness of first‐line psychosocial interventions. In such cases, AP drugs may be prescribed for a limited period and aimed primarily at alleviating psychological distress. Furthermore, current official EIP recommendations indicated that evidence for the effectiveness of AP therapy in CHR‐P adolescents is not enough to justify preventive pharmacological treatments and strongly discouraged AP use in adolescence (Raballo et al. 2022). In sum, our rate overall highlights the deep discrepancy between international guidelines' recommendations and real‐world practises, and is worthy of great clinical attention given APs' high potential for side effects in these youth populations.

As for clinical outcomes, the results of this examination showed that baseline AP exposure is associated with an increased 2‐year risk of new hospital admission and poorer functional recovery (especially in the high‐dose subsample). However, these findings may be attributable to the fact that CHR‐P individuals with more severe symptoms (and thus a greater need for higher doses of AP) are more prone to worse outcomes, such as a higher risk of hospital readmissions and impaired functional remission over time. In this sense, the adverse outcomes observed in the high‐dose AP subgroup may reflect the underlying severity and trajectory of their illness, rather than being direct consequences of high‐dose AP treatment itself. This interpretation is consistent with both clinical common sense (Raballo et al. 2024a) and the current understanding in the field (Raballo et al. 2025), suggesting that AP prescription at presentation, especially at high doses, is more likely a prognostic, ‘warning flag’ for poor outcomes (Raballo et al. 2023), rather than directly producing these unfavourable effects in young people at CHR‐P. Indeed, if AP exposure is related to more severe clinical and functional presentations perceived by the treating staff, this may reflect a more persistent and serious illness course, contributing to an increased risk for further hospital admission and poorer functional recovery over time. In this sense, CHR‐P individuals with AP prescription at entry could have a higher likelihood of new admission and poor longitudinal socio‐occupational functioning regardless of high‐dose AP treatment. Moreover, ongoing AP treatment at baseline could therefore clinically mask an already‐developed psychosis, making these cases incorrectly identified as CHR‐P subjects, rather than CHR‐P with a higher risk of transition (Raballo et al. 2025).

Notably, the results of this investigation also showed that AP‐naïve participants had an increased 2‐year risk of new self‐harm behaviours and a lower 2‐year incidence rate of PANSS symptomatic remission compared to the low‐dose AP subgroup. This finding was unexpected and leaves open another crucial question: could these findings be related to the prescription of low‐dose AP? Could low‐dose AP exposure in CHR‐P subjects reduce longitudinal risks of self‐harm and longitudinally promote symptom remission compared to the AP‐naïve subgroup? Could exposure to low doses of AP therefore improve the effectiveness of psychosocial EIP interventions compared to AP‐naïve individuals? However, these hypothetical positive effects did not appear to apply to baseline AP high doses. Could AP prescription be particularly useful in CHR‐P individuals with less clinical severity, potentially influencing the underlying trajectory of their disease? Moreover, as we found no longitudinal intergroup differences in terms of psychosis transition and CHR‐P criteria persistence, could AP prescription at entry also be effective in counteracting these unfavourable outcomes? In this respect, our ‘real‐world’ results contrast with past meta‐analytic evidence on an increased risk of psychosis conversion in CHR‐P individuals with baseline AP exposure (Raballo et al. 2024a). This may certainly be due to our relatively small sample size, and long‐term prevention of psychosis onset requires further investigations.

In sum, the results of this research suggest that low‐dose AP exposure can effectively induce symptom remission and a lower risk of self‐harm behaviour over time, but did not appear to influence functional recovery. Conversely, baseline APs at high doses appeared to be related to the worst functional prognosis, contributing to an increased risk of new hospital admission and poorer socio‐occupational functioning. In this regard, AP side effects (e.g., weight gain, sedation, motor disorders) could potentially contribute to affect daily functioning and quality of life in youths at CHR‐P, especially at high doses. This might adversely influence functional recovery, even if a good symptom improvement has been reached. Therefore, a careful, long‐term monitoring of APs in CHR‐P subjects is recommended.

## 5 Limitations

A first important limitation was related to the study design. Indeed, in this observational research, data were collected not for drug‐related purposes. Therefore, no information on adherence to treatment and AP side effects was examined. Further longitudinal investigations on longitudinal changes in AP use (such as novel adoption of drug) are needed. This way we would avoid directly inferring that APs themselves are the cause of the results we reported.

Additionally, follow‐up assessment of clinical outcomes in CHR‐P individuals was conducted using the PANSS, with symptomatic remission defined as a score of ≤ 3 on the eight items specified by the Remission in Schizophrenia Working Group (Andreasen et al. 2005). This definition may not be appropriate for CHR‐P populations, as a score of 3 on some PANSS positive items can indicate the presence of mild, vague, uncrystallized, and not tenaciously held psychotic symptoms (Kay et al. 1987). Whilst this is not strictly consistent with CHR‐P definitions, these PANSS remission criteria are widely accepted and used in psychosis research, including that on CHR‐P subjects (Yang et al. 2018). Our findings can therefore be replicated and discussed more easily in many other comparable CHR‐P samples, especially those primarily using the PANSS as a psychopathological assessment instrument. However, because the PANSS was originally designed for fully psychotic populations (Kay et al. 1987), outcome assessments, particularly those regarding clinical remission in CHR‐P individuals, should be supplemented using the CAARMS.

Moreover, no further examination on concomitant psychosocial and pharmacological treatments was performed. Additionally, this was a single‐centre study and may not be considered representative of all current ‘real‐world’ intervention protocols. Fourth, another weakness concerned our classification procedure. Indeed, we used the PDD/DDD ratio method. Therefore, our findings are comparable only with research using similar categorisation methodology. Finally, another limitation is related to our small sample size, particularly for the CHR‐P subgroups with baseline AP exposure. Future examination on larger CHR‐P samples is thus needed.

## 6 Conclusions

This observational investigation suggested that baseline AP exposure in young people at CHR‐P appeared not to be useful in preventing psychosis over time. Moreover, AP prescription at low doses showed to have some beneficial outcome effects. Specifically, it was longitudinally related to higher symptom remission and lower risk of self‐harm behaviour, whilst increasing the risk of new hospitalisation. Conversely, high‐dose AP exposure did not appear to provide any appreciable outcome result. Indeed, it was associated with a higher risk of hospital admission and poorer functional recovery. Therefore, APs should not always be the answer for all CHR‐P subjects and even for individuals with severe attenuated psychopathology, APs at high doses do not offer substantial benefits, along with their increased risk of side effects and functioning decline. Promoting personalised treatment strategies by integrating multidisciplinary interventions (including the prescription of AP at low doses) may be considered as a reasonable treatment option, although this requires further targeted investigations on larger, multi‐centre CHR‐P populations.

## Funding

The authors have nothing to report.

## Ethics Statement

Local relevant ethical approvals were obtained for the research (‘Area Vasta Emilia Nord’ ethics committee protocol no. 559/2020/OSS*/AUSLPR). This study was conducted in accordance with the Code of Ethics of the World Medical Association (1964 Declaration of Helsinki and its later amendments).

## Consent

All individuals and their parents (if minors) agreed to participate in the research and gave their written informed consent prior to their inclusion in the study.

## Conflicts of Interest

The authors declare no conflicts of interest.

## Supporting information


**Table S1:** Definitions of outcome parameters in the current study.
**Table S2:** Baseline comparisons on sociodemographic and clinical features amongst the three CHR‐P subgroups (*n* = 182).
**Figure S1:** Prescribing pattern of AP medication in the total CHR‐P sample (*n* = 182).
**Figure S2:** Survival functions for time‐to‐event outcome incidence rates amongst the three CHR‐P subgroups across the 2‐year follow‐up period.
**Figure S3:** Estimated plots: binary logistic regression analysis results for not time‐to‐event outcome variables in the three CHR‐P subgroups across the 2‐year follow‐up period.

## Data Availability

The data that support the findings of this study are available on request from the corresponding author. The data are not publicly available due to privacy or ethical restrictions.

## References

[eip70167-bib-0001] Addington, J. , D. Addington , S. Abidi , T. Raedler , and G. Remington . 2017. “Canadian Treatment Guidelines for Individuals at Clinical High Risk of Psychosis.” Canadian Journal of Psychiatry 62: 656–661. 10.1177/0706743717719895. 28730848 PMC5593244

[eip70167-bib-0002] Andreasen, N. C. , W. T. Carpenter Jr. , J. M. Kane , R. A. Lasser , S. R. Marder , and D. R. Weinberger . 2005. “Remission in Schizophrenia: Proposed Criteria and Rationale for Consensus.” American Journal of Psychiatry 162: 441–449. 10.1176/appi.ajp.162.3.441. 15741458

[eip70167-bib-0003] Armstrong, R. A. 2014. “When to Use the Bonferroni Correction.” Ophthalmic and Physiological Optics 34: 502–508. 10.1111/opo.12131. 24697967

[eip70167-bib-0004] Barnes, T. R. , R. Drake , C. Paton , et al. 2020. “Evidence‐Based Guidelines for the Pharmacological Treatment of Schizophrenia: Updated Recommendations From the British Association for Psychopharmacology.” Journal of Psychopharmacology 34: 3–78. 10.1177/0269881119889296. 31829775

[eip70167-bib-0005] Biancalani, A. , M. Occhionero , E. Leuci , et al. 2025. “Disorganization in Individuals at Clinical High Risk for Psychosis: Psychopathology and Treatment Response.” European Archives of Psychiatry and Clinical Neuroscience 275: 921–935. 10.1007/s00406-024-01855-3. 38914855 PMC11947017

[eip70167-bib-0006] Buchanan, R. W. 2007. “Persistent Negative Symptoms in Schizophrenia: An Overview.” Schizophrenia Bulletin 33: 1013–1022. 10.1093/schbul/sbl057. 17099070 PMC2632326

[eip70167-bib-0007] Carrión, R. E. , B. A. Cornblatt , C. Z. Burton , et al. 2016. “Personalized Prediction of Psychosis: External Validation of the NAPLS‐2 Psychosis Risk Calculator With the EDIPPP Project.” American Journal of Psychiatry 173: 989–996. 10.1176/appi.ajp.2016.15121565. 27363511 PMC5048503

[eip70167-bib-0008] Catalan, A. , G. de Salazar Pablo , J. Vaquerizo Serrano , et al. 2020. “Annual Research Review: Prevention of Psychosis in Adolescents Systematic Review and Meta‐Analysis of Advances in Detection, Prognosis and Intervention.” Journal of Child Psychology and Psychiatry 62: 657–673. 10.1111/jcpp.13322. 32924144

[eip70167-bib-0009] Catalano, F. , E. Leuci , E. Quattrone , et al. 2025. “Clinical High Risk for Psychosis and Service Disengagement: Incidence and Predictors Across 2 Years of Follow‐Up.” Early Intervention in Psychiatry 19: e13599. 10.1111/eip.13599. 39034609 PMC11730404

[eip70167-bib-0010] Di Lisi, A. , E. Leuci , E. Quattrone , et al. 2024. “Obsessive‐Compulsive Symptoms in Individuals at Clinical High Risk for Psychosis: A 2‐Year Longitudinal Study.” Schizophrenia Research 274: 11–20. 10.1016/j.schres.2024.09.005. 39244946

[eip70167-bib-0011] Di Lisi, A. , S. Pupo , M. Menchetti , and L. Pelizza . 2024. “Antipsychotic Treatment in People at Clinical High Risk for Psychosis: A Narrative Review of Suggestions for Clinical Practice.” Journal of Clinical Psychopharmacology 44: 502–508. 10.1097/JCP.0000000000001891. 39250139 PMC11460766

[eip70167-bib-0012] German Association for Psychiatry, Psychotherapy, and Psychosomatics (GAPPP) . 2019. German Association for Psychiatry, Psychotherapy and Psychosomatics Guideline for Schizophrenia. GAPPP Publishing.

[eip70167-bib-0013] Goel, M. K. , P. Khanna , and J. Kishore . 2010. “Understanding Survival Analysis: Kaplan‐Meier Estimate.” International Journal of Ayurveda Research 1: 274–278. 10.4103/0974-7788.76794. 21455458 PMC3059453

[eip70167-bib-0014] Gruppo di Lavoro Regionale (GLR) . 2024. «Linee di indirizzo per la promozione della salute e del benessere nelle persone alla prima manifestazione psicotica o ad alto rischio di psicosi.» Centro stampa della Regione Emilia‐Romagna. https://salute.regione.emilia‐romagna.it/salute‐mentale/percorsi‐di‐cura/esordi.

[eip70167-bib-0015] Haas, S. S. , R. Ge , I. Agartz , et al. 2024. “Normative Modeling of Brain Morphometry in Clinical High Risk for Psychosis.” JAMA Psychiatry 81: 77–88. 10.1001/jamapsychiatry.2023.3850. 37819650 PMC10568447

[eip70167-bib-0016] Harris, J. K. 2021. “Primer on Binary Logistic Regression.” Family Medicine and Community Health 9, no. Suppl. 1: e001290. 10.1136/fmch-2021-001290. 34952854 PMC8710907

[eip70167-bib-0017] IBM Corporation (IBM Corp.) . 2021. IBM SPSS Statistics for Windows, Version 28.0. IBM Corp. Press.

[eip70167-bib-0018] Kay, S. R. , A. Fiszbein , and L. A. Opler . 1987. “The Positive and Negative Syndrome Scale (PANSS) for Schizophrenia.” Schizophrenia Bulletin 13: 261–276. 10.1093/schbul/13.2.261. 3616518

[eip70167-bib-0019] Leucht, S. , M. Samara , S. Heres , and J. M. Davis . 2016. “Dose Equivalents for Antipsychotic Drugs: The DDD Method.” Schizophrenia Bulletin 42, no. Suppl. 1: S90–S94. 10.1093/schbul/sbv167. 27460622 PMC4960429

[eip70167-bib-0020] Liu, C. C. , M. C. Lai , C. M. Liu , et al. 2011. “Follow‐Up of Subjects With Suspected Pre‐Psychotic State in Taiwan.” Schizophrenia Research 126: 65–70. 10.1016/j.schres.2010.10.028. 21112187

[eip70167-bib-0021] Pancheri, P. , R. Brugnoli , L. Carilli , R. Delle Chiaie , P. L. Marconi , and R. M. Petrucci . 1995. “Valutazione dimensionale della sintomatologia schizofrenica: validazione della versione italiana della Scala per la valutazione dei Sintomi Positivi e Negativi (PANSS).” Journal of Psychopathology 1: 60–75.

[eip70167-bib-0022] Paterlini, F. , L. Pelizza , G. Galli , et al. 2019. “Interrater Reliability of the Authorized Italian Version of the Comprehensive Assessment of at‐Risk Mental States (CAARMS‐ITA).” Journal of Psychopathology 25: 24–28. https://old.jpsychopathol.it/wp‐content/uploads/2019/02/05_Raballo‐1.pdf.

[eip70167-bib-0024] Pelizza, L. , A. Di Lisi , E. Leuci , et al. 2025. “Baseline Exposure to Antipsychotic Medication in Young People at Clinical High Risk for Psychosis: A 2‐Year Italian Follow‐Up Study.” Human Psychopharmacology 40: e70003. 10.1002/hup.70003. 39962036 PMC11832455

[eip70167-bib-0023] Pelizza, L. , A. Di Lisi , E. Leuci , et al. 2025. “Subgroups of Clinical High Risk for Psychosis Based on Baseline Antipsychotic Exposure: Clinical and Outcome Comparisons Across a 2‐Year Follow‐Up Period.” Schizophrenia Bulletin 51: 432–445. 10.1093/schbul/sbae029. 38551332 PMC11908853

[eip70167-bib-0025] Pelizza, L. , E. Leuci , D. Maestri , et al. 2022. “Longitudinal Persistence of Negative Symptoms in Young Individuals With First Episode Schizophrenia: A 24‐Month Multi‐Modal Program Follow‐Up.” Nordic Journal of Psychiatry 76: 530–538. 10.1080/08039488.2021.2015431. 34936855

[eip70167-bib-0026] Pelizza, L. , E. Leuci , E. Quattrone , et al. 2024. “Baseline Antipsychotic Prescription and Short‐Term Outcome Indicators in Individuals at Clinical High‐Risk for Psychosis: Findings From the Parma at‐Risk Mental States (PARMS) Program.” Early Intervention in Psychiatry 18: 71–81. 10.1111/eip.13434. 37194411

[eip70167-bib-0027] Pelizza, L. , E. Leuci , E. Quattrone , G. Paulillo , and P. Pellegrini . 2023. “The “Parma at‐Risk Mental States” (PARMS) Program: General Description and Process Analysis After 5 Years of Clinical Activity.” Early Intervention in Psychiatry 17: 625–635. 10.1111/eip.13399. 36639137

[eip70167-bib-0028] Pelizza, L. , F. Paterlini , S. Azzali , et al. 2019. “The Approved Italian Version of the Comprehensive Assessment of At‐Risk Mental States (CAARMS‐ITA): Field Test and Psychometric Features.” Early Intervention in Psychiatry 13: 810–817. 10.1111/eip.12669. 29696795

[eip70167-bib-0029] Poletti, M. , L. Pelizza , A. Preti , and A. Raballo . 2024. “Clinical High‐Risk for Psychosis (CHR‐P) Circa 2024: Synoptic Analysis and Synthesis of Contemporary Treatment Guidelines.” Asian Journal of Psychiatry 100: 104142. 10.1016/j.ajp.2024.104142. 39083954

[eip70167-bib-0030] Preti, A. , A. Raballo , A. Meneghelli , et al. 2022. “Antipsychotics Are Related to Psychometric Conversion to Psychosis in Ultra‐High‐Risk Youth.” Early Intervention in Psychiatry 16: 342–351. 10.1111/eip.13158. 33951751 PMC9291179

[eip70167-bib-0031] Raballo, A. , M. Poletti , and W. T. Carpenter . 2019. “Rethinking the Psychosis Threshold in Clinical High Risk.” Schizophrenia Bulletin 45: 1–2. 10.1093/schbul/sby149. 30339224 PMC6293213

[eip70167-bib-0032] Raballo, A. , M. Poletti , and A. Preti . 2020. “Meta‐Analyzing the Prevalence and Prognostic Effect of Antipsychotic Exposure in Clinical High‐Risk (CHR): When Things Are Not What They Seem.” Psychological Medicine 50: 2673–2681. 10.1017/S0033291720004237. 33198845

[eip70167-bib-0033] Raballo, A. , M. Poletti , and A. Preti . 2021. “Antipsychotic Treatment in Clinical High Risk for Psychosis: Protective, Iatrogenic or Further Risk Flag?” Australian and New Zealand Journal of Psychiatry 55: 442–444. 10.1177/0004867420984836. 33426910

[eip70167-bib-0034] Raballo, A. , M. Poletti , and A. Preti . 2022. “Editorial Perspective: Psychosis Risk in Adolescence ‐ Outcomes, Comorbidity, and Antipsychotics.” Journal of Child Psychology and Psychiatry 63: 241–244. 10.1111/jcpp.13438. 34085715

[eip70167-bib-0035] Raballo, A. , M. Poletti , and A. Preti . 2023. “The Temporal Dynamics of Transition to Psychosis in Individuals at Clinical High‐Risk (CHR‐P) Shows Negative Prognostic Effects of Baseline Antipsychotic Exposure: A Meta‐Analysis.” Translational Psychiatry 13: 112. 10.1038/s41398-023-02405-6. 37019886 PMC10076303

[eip70167-bib-0036] Raballo, A. , M. Poletti , and A. Preti . 2024a. “ENIGMA Brain Morphometry in CHR‐P: Risk Enrichment and Antipsychotics.” JAMA Psychiatry 81: 427–428. 10.1001/jamapsychiatry.2023.5611. 38353961

[eip70167-bib-0037] Raballo, A. , M. Poletti , and A. Preti . 2024b. “Baseline Antipsychotic Dose and Transition to Psychosis in Individuals at Clinical High Risk: A Systematic Review and Meta‐Analysis.” JAMA Psychiatry 81: 727–730. 10.1001/jamapsychiatry.2024.0178. 38506802 PMC10955337

[eip70167-bib-0038] Raballo, A. , M. Poletti , and A. Preti . 2025. “Increasing Conceptual Clarity and Confounders Identification: A Pragmatic Way to Enhance Prognostic Precision in ENIGMA Clinical High Risk for Psychosis (CHR‐P).” Molecular Psychiatry 30: 3319–3320. 10.1038/s41380-025-02948-8. 40097609

[eip70167-bib-0039] Ricci, C. , E. Leuci , E. Quattrone , et al. 2024. “Persistent Negative Symptoms in Young People at Clinical High Risk of Psychosis Treated With an Italian Early Intervention Program: A Longitudinal Study.” European Archives of Psychiatry and Clinical Neuroscience 274: 1311–1326. 10.1007/s00406-024-01808-w. 38668766 PMC11362215

[eip70167-bib-0040] Roh, D. , J. G. Chang , C. H. Kim , H. S. Cho , S. K. An , and Y. C. Jung . 2014. “Antipsychotic Polypharmacy and High‐Dose Prescription in Schizophrenia: A 5‐Year Comparison.” Australian and New Zealand Journal of Psychiatry 48: 52–60. 10.1177/0004867413488221. 23671214

[eip70167-bib-0041] Schennach‐Wolff, R. , M. Jäger , F. Seemüller , et al. 2009. “Defining and Predicting Functional Outcome in Schizophrenia and Schizophrenia Spectrum Disorders.” Schizophrenia Research 113: 210–217. 10.1016/j.schres.2009.05.032. 19560901

[eip70167-bib-0042] Taipale, H. , A. Tanskanen , C. U. Correll , and J. Tiihonen . 2022. “Real‐World Effectiveness of Antipsychotic Doses for Relapse Prevention in Patients With First‐Episode Schizophrenia in Finland: A Nationwide, Register‐Based Cohort Study.” Lancet Psychiatry 9: 271–279. 10.1016/S2215-0366(22)00015-3. 35182475

[eip70167-bib-0043] Tognin, S. , S. Vieira , D. Oliver , et al. 2025. “PSYSCAN Multi‐Centre Study: Baseline Characteristics and Clinical Outcomes of the Clinical High Risk for Psychosis Sample.” Schizophrenia 11: 66. 10.1038/s41537-025-00598-x. 40246889 PMC12006469

[eip70167-bib-0044] World Health Organization Collaborating Centre for Drug Statistics Methodology (WHO) . 2002. Guidelines for Anatomical Therapeutic Chemical (ATC) Classification and DDD Assignment. WHO Publishing.

[eip70167-bib-0045] Yang, Z. , K. Lim , M. Lam , R. Keefe , and J. Lee . 2018. “Factor Structure of the Positive and Negative Syndrome Scale (PANSS) in People at Ultra‐High Risk (UHR) for Psychosis.” Schizophrenia Research 201: 85–90. 10.1016/j.schres.2018.05.024. 29804925

[eip70167-bib-0046] Yung, A. R. , H. P. Yuen , P. D. McGorry , et al. 2005. “Mapping the Onset of Psychosis: The Comprehensive Assessment of at‐Risk Mental States.” Australian and New Zealand Journal of Psychiatry 39: 964–971. 10.1080/j.1440-1614.2005.01714.x. 16343296

[eip70167-bib-0050] Zeng, J. , A. Raballo , R. Gar , et al. 2022. “Antipsychotic Exposure in Clinical High Risk of psychosis: Empirical Insight from a Large Cohort Study.” Journal of Clinical Psychiatry 83: 21m14092. 10.4088/JCP.21m14092. 35324095

[eip70167-bib-0047] Zhang, T. , A. Raballo , J. Zeng , et al. 2022. “Antipsychotic Prescription, Assumption and Conversion to Psychosis: Resolveng Missing Clinical Links to Optimize Prevention Through Precision.” Schizophrenia 8: 48. 10.1038/s41537-022-00254-8. 35853891 PMC9261109

[eip70167-bib-0048] Zhang, T. , J. Wang , L. Xu , et al. 2022. “Further Evidence That Antipsychotic Medication Does Not Prevent Long‐Term Psychosis in Higher‐Risk Individuals.” European Archives of Psychiatry and Clinical Neuroscience 272: 591–602. 10.1007/s00406-021-01331-2. 34536114

[eip70167-bib-0049] Zhu, Y. , N. Maikusa , J. Radua , et al. 2024. “Using Brain Structural Neuroimaging Measures to Predict Psychosis Onset for Individuals at Clinical High‐Risk.” Molecular Psychiatry 29: 1465–1477. 10.1038/s41380-024-02426-7. 38332374 PMC11189817

