# Supplementary material for: Exposure to Antipsychotics in Youths at Clinical High Risk for Psychosis: Low VS High Doses and Their Relevance for Clinical Outcomes
Source: Early Interv Psychiatry. 2026 Mar 22;20(4):e70167. doi: 10.1111/eip.70167 (PMC13006195; doi:10.1111/eip.70167)
Supplement: Supplementary file 1 — Table S1: Definitions of outcome parameters in the current study. Table S2: Baseline comparisons on sociodemographic and clinical features amongst the three CHR‐P subgroups (n = 182). Figure S1: Prescribing pattern of AP medication in the total CHR‐P sample (n = 182). Figure S2: Survival functions for time‐to‐event outcome incidence rates amongst the three CHR‐P subgroups across the 2‐year follow‐up period. Figure S3: Estimated plots: binary logistic regression analysis results for not time‐to‐event outcome variables in the three CHR‐P subgroups across the 2‐year follow‐up period. [file EIP-20-0-s001.doc]

Table S1. Definitions of outcome parameters in the current study.

| - Service disengagement = complete lack of contact or untraceable for at least 3 months despite a need of treatment, counted from the date of the last face-to-face meeting with the clinical staff (Pelizza et al., 2023a). - Suicide attempt = potentially injurious, self-inflicted behavior without a fatal outcome for which there was (explicit or implicit) evidence of intent to die (Silverman et al., 2007). - Self-harm behavior = acts of deliberate self-harm or intoxication with alcohol or drugs, but where there was no clear intention to die (Silverman et al., 2007). - Functional recovery = return to work/school (Santesteban-Echarri et al., 2017). - Current suicidal ideation = “at least occasional suicidal thoughts without intent or specific plan, or the patients feel they would be better off dead”, as indicated by a Brief Psychiatric Rating Scale (BPRS) item 4 score of ≥3 (Shafer et al., 2017). |
| --- |

**References**

- Pelizza L, Leuci E, Quattrone E, Azzali S, Pupo S, Paulillo G, Pellegrini P, Menchetti M. 2023. Short-term disengagement from early intervention service for first-episode psychosis: findings from the “Parma Early Psychosis” program. Soc Psychiatry Psychiatr Epidemiol, 59, 1201-1213. <https://doi.org/10.1007/s00127-023-02564-3>.
- Santesteban-Echarri O, Paino M, Rice S, González-Blanch C, McGorry P, Gleeson J, Alvarez-Jimenez M. 2017. Predictors of functional recovery in first-episode psychosis: a systematic review and meta-analysis of longitudinal studies. Clin Psychol Rev, 58, 59-75. <https://doi.org/10.1016/j.cpr.2017.09.007>.
- Silverman MM, Berman AL, Sanddal ND, O’carroll PW, Joiner TE. 2007. Rebuilding the tower of Babel: a revised nomenclature for the study of suicide and suicidal behaviors - Part 2: suicide-related ideations, communications, and behaviors. Suicide Life Threat Behav, 37, 264-277. <https://doi.org/10.1521/suli.2007.37.3.264>.
- Shafer A, Dazzi F, Ventura J. Factor structure of the Brief Psychiatric Rating Scale - Expanded (BPRS-E) in a large hospitalized sample. J Psychiatr Res. 2017; 93: 79-86. <https://doi.org/10.1016/j.jpsychires.2017.05.011>.

Table S2 – Baseline comparisons on sociodemographic and clinical features among the three CHR-P subgroups (n=182).

| Variables | AP-naïve | Low dose | High dose | X^2^/H | p | Post-hoc comparisons |
| --- | --- | --- | --- | --- | --- | --- |
| Gender (males)  Age at entry  Education (in years)  Ethnic group (White)  DUI (in weeks)  Substance abuse  *CHR-P criteria*  APS  BLIPS  VG  PANSS total score  SOFAS score | 46 (51.6%)  18.24±3.77  11.13±2.32  79 (89.8%)  51.67±53.95  13 (14.6%)  77 (86.5%)  7 (7.9%)  5 (5.6%)  68.77±17.51  49.94±9.82 | 29 (48.3%)  20.68±3.38  11.42±2.60  54 (90.0%)  38.10±43.86  11 (18.3%)  41 (68.3%)  15 (25.0%)  4 (6.7%)  69.15±13.37  48.78±6.65 | 16 (48.4%)  21.06±3.36  11.97±2.72  26 (81.2%)  52.21±48.45  8 (24.2%)  24 (72.7%)  8 (24.2%)  1 (3.1%)  78.22±20.72  45.70±7.60 | .198  21.484  4.240  1.897  3.654  1.577  7.569  9.406  .344  4.311  6.985 | .906  **.001**  .120  .387  .161  .454  **.023**  **.009**  .842  .116  **.030** | -  AP-naïve < Low dose = High dose  -  -  -  -  AP-naïve > Low dose  AP-naïve < High dose  -  -  AP-naïve > High dose |

Note. CHR-P = Clinical High Risk for Psychosis; AP = Antipsychotic; AP-naive = CHR-P individuals without baseline AP exposure; Low dose = CHR-P individuals with baseline low dose AP exposure (i.e., PDD/DDD ratio <0.6); High dose = CHR-P individuals with baseline high dose AP exposure (i.e., with a PDD/DDD ratio ≥0.6); PDD = Prescribed Daily Dose of AP; DDD = Defined Daily Dose of AP; DUI = Duration of Untreated Illness; APS = Attenuated Psychotic Symptoms; BLIPS = Brief Limited Intermittent Psychotic Symptoms; VG = Vulnerability Group; PANSS = Positive And Negative Syndrome Scale; SOFAS = Social And Occupational Functioning Assessment Scale. Frequencies (and percentages) and mean ± standard deviations are reported. Chi-square (X^2^) and Kruskal-Wallis (H) test values are reported. Statistically significant p values are in bold. Bonferroni corrected p values are reported.

Figure S1 - Prescribing pattern of AP medication in the total CHR-P sample (n=182).

5

Service disengagement

21

Service disengagement

| 89 (48.9%)  AP-naive  60 (33.0%)  Low-dose  33 (18.1%)  High-dose  85  AP-naive  60  Low-dose  32  High-dose  70  AP-naive  54  Low-dose  29  High-dose |
| --- |

Note. AP = Antipsychotic; CHR-P = Clinical High Risk for Psychosis; AP naive = CHR-P participants without baseline AP prescription; CHR-P/AP+ = CHR-P participants with baseline AP prescription; PDD = Prescribed daily dose of AP; DDD = Defined daily dose of AP; Low-dose = CHR-P/AP+ individuals with PDD/DDD ratio < 0.6 (i.e., less than 0.6 defined daily doses per day, corresponding to 3.0 mg per day equivalent risperidone); High-dose = CHR-P/AP+ individuals with a PDD/DDD ratio ≥ 0.6; T0 = baseline assessment; T1 = 1-year assessment time; T2 = 2-year assessment time.

Figure S2 – Survival functions for time-to-event outcome incidence rates among the three CHR-P subgroups across the 2-year follow-up period.


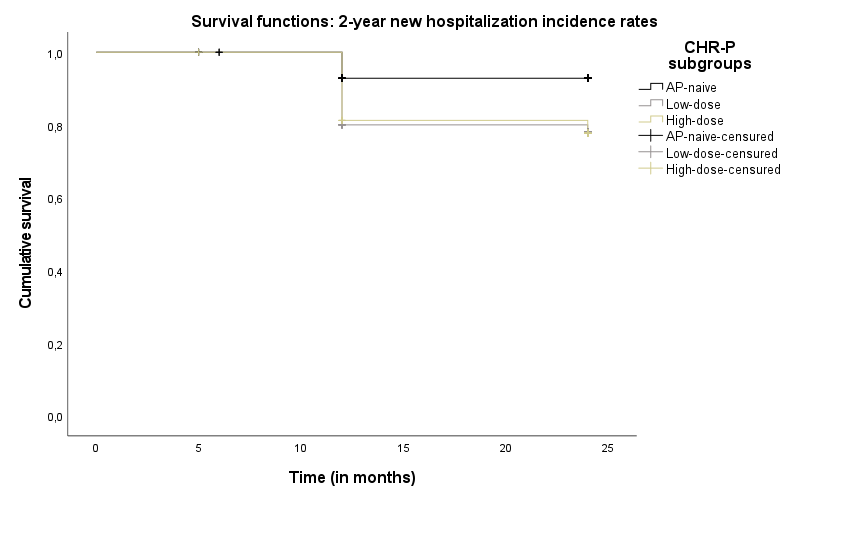


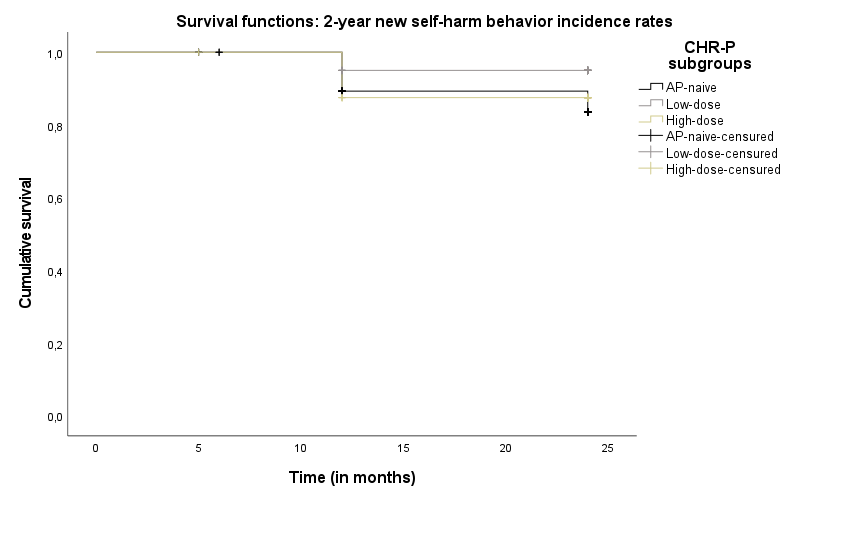


Note. AP = Antipsychotic; CHR-P = Clinical High Risk for Psychosis; AP naive = CHR-P participants without baseline AP prescription; CHR-P/AP+ = CHR-P participants with baseline AP prescription; PDD = Prescribed daily dose of AP; DDD = Defined daily dose of AP; Low-dose = CHR-P/AP+ individuals with PDD/DDD ratio < 0.6 (i.e., less than 0.6 defined daily doses per day, corresponding to 3.0 mg per day equivalent risperidone); High-dose = CHR-P/AP+ individuals with a PDD/DDD ratio ≥ 0.6.

Figure S3 – Estimated plots: binary logistic regression analysis results for not time-to-event outcome variables in the three CHR-P subgroups across the 2-year follow-up period.


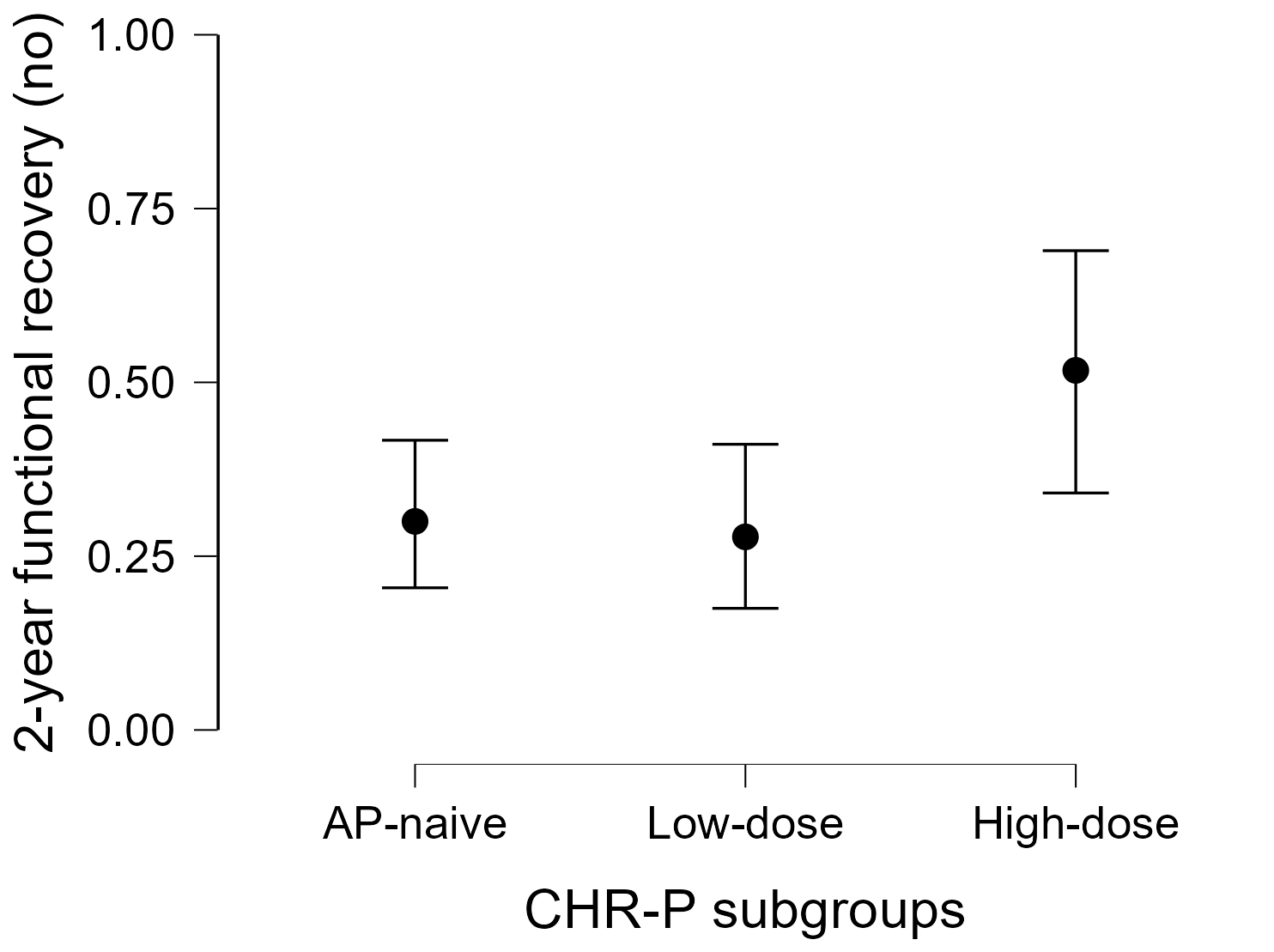


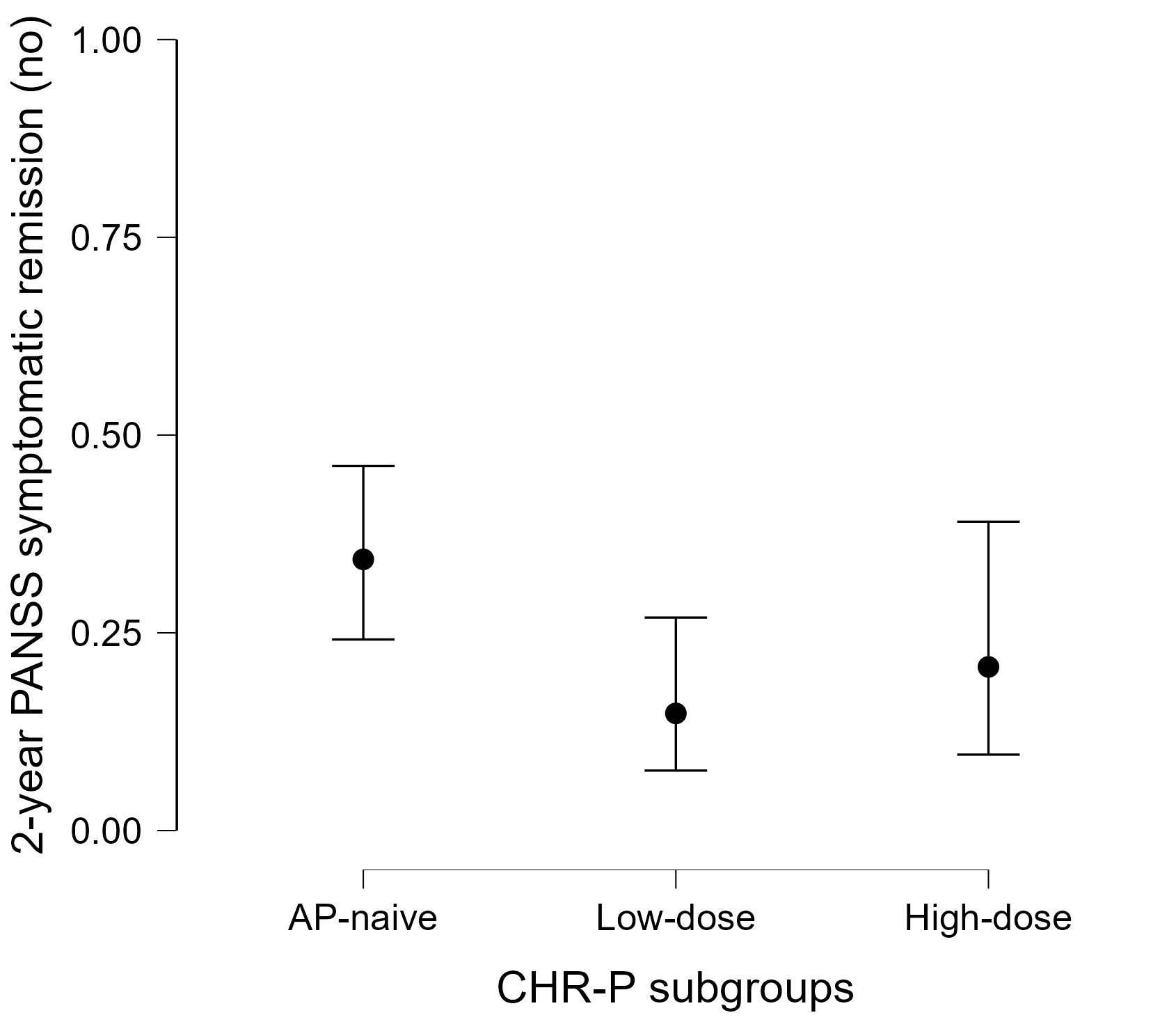


Note. AP = Antipsychotic; CHR-P = Clinical High Risk for Psychosis; AP naive = CHR-P participants without baseline AP prescription; PDD = Prescribed daily dose of AP; DDD = Defined daily dose of AP; Low-dose = CHR-P/AP+ individuals with PDD/DDD ratio <0.6 (i.e., less than 0.6 defined daily doses per day, corresponding to 3.0 mg per day equivalent risperidone); High-dose = CHR-P/AP+ individuals with a PDD/DDD ratio ≥0.6; PANSS = Positive And Negative Syndrome Scale.
